# Market Care and Custody: A Health Policy Analysis of Incarceration and Long-Term Care Systems in the U.S.

**DOI:** 10.3390/jmahp14030039

**Published:** 2026-07-17

**Authors:** Travis W. Milburn, Iffath Unissa Syed

**Affiliations:** 1 School of Criminology, Criminal Justice, and Legal Studies, Grand Valley State University, Grand Rapids, MI 49504, USA; milburtr@gvsu.edu; 2 Department of Health Policy and Administration, The Pennsylvania State University, Sharon, PA 16146, USA

**Keywords:** marketization, public health policy, privatization, neoliberalism, incarceration, long-term care, profitization, commercial determinants of health

## Abstract

The United States has one of the world’s largest criminal justice systems, with nearly 5.5 million people under correctional supervision and almost 2 million incarcerated. This scale of confinement, coupled with the rise of privatization across correctional and related services, reflects a broader neoliberal trend in public governance. This paper explores the consequences of privatization and marketization of the U.S. criminal justice system—particularly the proliferation of private prisons and immigrant detention centers—and draws parallels to the consequences of privatization of health and social care, especially long-term care (LTC). Both systems reveal shared logics of marketization that prioritize profit maximization, efficiency, and cost-cutting at the expense of care, justice, and equity. Relying on interdisciplinary perspectives from public health and criminology, this paper situates private corrections within the health policy framework of the Commercial Determinants of Health (CDoH), arguing that privatized carceral institutions not only harm incarcerated individuals but also endanger workers, families, and surrounding communities through systemic under-resourcing, precarious labor conditions, and structural violence. By comparing the private, for-profit prison industry with private for-profit LTC systems, we illustrate how these structures have commodified both care and correctional systems. These findings suggest that privatization within carceral and care sectors perpetuates health inequities and reinforces cycles of racial, gendered, and economic disadvantage. Accordingly, this paper calls for strengthening publicly held models and a reassertion of public accountability and interdisciplinary collaboration to restore social justice, health, and human dignity as central organizing principles of both systems for residents, workers, their families, and communities.

## 1. Introduction and Background

The United States criminal justice system is renowned for its scope in detention and control and the number of individuals with whom it comes into contact and supervises at any given time. Between 1990 and 2017, a total of 376,923,113 arrests were made [1,2]. Though the total number of arrests had decreased in recent years, they seem to be on the rise: in 2024 alone, 7.5 million, arrests were made [3].

Beyond arrest, at year-end 2022, almost 5.5 million people were under correctional supervision of some kind by an adult correctional system, a figure that includes people in jails, prisons, or on probation or parole. About 1.8 million people were incarcerated in the U.S. as of 2024 (a slight increase from 2022). Of these residents, 659,000 people were in locally operated jails which are used for pre-conviction detention and shorter sentences of less than one-year of incarceration and are, therefore, temporary in nature, and used in bookings while waiting for trials, transfer of inmates [4]. In addition, over 1.25 million people are in prisons which operate mostly at the state level and house individuals after conviction for sentences of a year or more [4]. Significantly, the incarceration rate in the United States is one of the highest globally at 557 persons per 100,000 population in 2024 [4]. Beyond incarceration, an additional 3.6 million individuals are being supervised via probation or parole in the community [5]. Taken together, the U.S. criminal justice system oversees a massive number of people at any given time.

Researchers have described these trends as very troubling, and for those from minority communities who have experienced systematic forms of discrimination, racialization, and structural violence, perhaps even frightening. In fact, one in three Black men in the United States is incarcerated at some point in their lives [6]. Ironically, during the height of the incarceration boom in the U.S., incarceration rates significantly outpaced population growth:

“Between 1980 and 2012, the total number of state and local prisoners in the United States rose from 501,886 to 2,228,400-a 344% increase, while the U.S. population grew [at] the same time only from 226.5 million to 313 million-a 38% increase”.

[7] (p. 413)

In addition to the number of people residing in the prison system, approximately 400,000 employees work in carceral facilities as corrections officers, healthcare providers, administrators, educators, volunteers, and so forth [8]. Often overlooked within the corrections ecosystem is the role of privatization, which exists in each of the areas described above, as well as within other spaces in the criminal justice system.

In this paper, we theoretically synthesize and explore the concepts and consequences of privatization and marketization, and include perspectives from the fields of public health policy and criminology. In particular, we draw on parallel themes found between the privatization of criminal justice and the privatization of health and social care, bringing together these two interdisciplinary areas. The rationale for bringing together an examination of these seemingly different domains is twofold. First, the field of criminal justice and the field of health/social care include three sets of actors and organizations; the first consists of employees/workers, the second consists of residents (ironically, a similar number of residents (1.3 million) are in LTC facilities [9], and finally the third consists of structures that generate capital in exchange for offering their services.

To date, there has been no work bringing together and theorizing the conditions in these two distinct yet similarly functioning organizational contexts consisting of growing entanglement with neoliberal, often private enterprise.

As noted in scholarly discourse, various areas of social policies have become ever more privatized and include areas such as public benefits management and welfare services, as well as long-term care [10,11]. Regarding criminal justice services, reliance on neoliberal (and often private) entities is also widespread and includes spaces like drug/alcohol treatment, housing, personnel training, transportation or correctional populations, educational programming [12], jails [13] and, most notably for our purposes, prisons [11,12,13,14]. Although we discuss the detention of immigrants as an aspect of the for-profit prison system through this lens of neoliberalism, it is beyond the scope of this paper to provide the policies, procedures, executive actions, and administrative responses on the topic of immigration detention. Below, we begin with the history and function of privatization with a focus on neoliberal (and often private) prisons.

## 2. History and Function of For-Profit Prisons and Long-Term Care

Neoliberalism is a “political and economic valorization of market forces and orientation to market ‘freedoms’, which is both framed and reproduced by ideological […] strategies emphasizing individualism, competition and restrictions on state intervention” [15] (p. 162). There is an abundance of literature discussing the damaging effects of neoliberal policies, systems, and structures. On the other hand, marketization involves restructuring and the introduction of markets and market forces that transform agencies and services [16]. These are not singular processes; they are varied and uneven [16].

However, we note that the literature often discusses and conflates the aforementioned terms neoliberalism and marketization with privatization.

Privatization in a criminal justice context has been defined as a situation wherein “for-profit companies generate money from their engagement with local, state, and federal judicial and correctional systems” [11] (p. 351). We note that this latter definition is flexible. First, in capitalist societies, generating profits is inherently seen as a good objective and can be achieved by privately held or publicly held entities. For-profit entities can contract with governments to house residents, and they can be either publicly listed and owned (on the stock market) or privately owned companies. For the latter, private entities are owned by individuals, families, or private equity shareholders. Often, when researchers refer to private prisons or private LTC, it excludes government-owned and government-operated facilities or may include government-owned but privately operated/managed facilities (i.e., outsourced). For the purpose of this paper, and in order to be consistent with the meanings of private and public entities in the criminal justice and LTC literature, we opt to use terminology such as private/neoliberal or market-oriented/investor-driven institutions.

It is noteworthy that for-profit (and often private) entities have been used widely in U.S. criminal justice for decades and span across the system. While the process of generating revenues is not problematic, how profits are maximized and what is done with those revenues is crucial. For-profit public entities (e.g., state-owned) often redistribute revenues and reinvest them, often used for budget line items like hiring/human resources, investment in infrastructure, and so forth. However, for-profit private entities, which are often publicly listed (meaning that they have publicly traded shares in an open market), will redistribute profits to shareholders [17]. Financial managers often act in the best interests of these stockholders/shareholders by taking actions that increase the value of the stock, which are tied to managerial compensation structures and financial performance in general [18]. However, in doing so, management may prioritize revenue-generation and increasing stock value at the expense of worker contexts, among other things.

In the area of corrections, privatization, neoliberalization, and marketization occur in both community supervision and in-custody corrections (i.e., jails and prisons). These entities provide services carried out as part of community supervision, including programming, and in some places, they also are directly involved in supervision [12]. Scholars describe how a variety of entities profit from increased incarceration in the U.S. over the last several decades as the “prison-industrial complex” (PIC). This term helps elucidate the various economic, organizational, and bipartisan political interests that have resulted in an increase in use of and spending on imprisonment. A major characteristic of this trend is the supplanting of a public service mentality with one fixated on increased profit-seeking through sweeping crime policy and sentencing changes [19,20]. Importantly, the establishment and furtherance of the PIC is more firmly connected to political, social, and ideological forces than to criminality itself [20].

One significant component of the PIC is the process of neoliberalization and marketization in corrections, which has caused a blurring of interests. Tantamount to this trend is the prison boom [19] which continues today. The practice of allocating public funds to contract corporations to manage incarcerated populations has existed since the 1980s [11]. There are two entities that have dominated the prison landscape since its inception—GEO Group and CoreCivic (formerly Corrections Corporation of America) [21]. These companies, among others, provided relief for correctional systems as prison populations began to grow dramatically, reaching their height over a decade ago [21,22]. The entities (major institutional holders) that are the largest shareholders in both GEO Group and CoreCivic include private equity firms and major fund managers such as Black Rock Inc., Vanguard Group Inc., Pentwater Capital Management LP, River Road Asset Management, LLC, Cooper Creek Partners LLC, State Street Corporation, and Goldman Sachs Group Inc., among others [23,24,25].

Since 2000, there has been a 5% increase in the number of people housed in these private, neoliberal (and often corporate) prison systems, which includes both state and federal prison residents [22]. More specifically, there was a 165% growth in private residency of federal inmates and a 24% increase in state prisons from 2000 to 2016 [26]. At year-end 2022, 8% of all prison inmates (or 90,873 people) resided in a private, corporately owned facility [22]. About 77,500 individuals resided in privately operated state facilities (a 3% increase from 2021) and 6200 individuals were housed in U.S. Bureau of Prisons facilities (a 57% decrease from 2021). State prison systems house the most individuals in the U.S.—986,400 of the 1,145,000 individuals imprisoned in the U.S. [5], making the increased use of private facilities in this space significant. Importantly, private prison companies mentioned previously make a large portion of their revenue from immigrant detention [21] which is excluded from the figures above.

Though the annual changes in the percentage of correctional facility residents in private facilities during the first few years of the 2020s may appear to suggest decreased reliance on the use of privatization, a variety of signs indicate an impending increase in the use of private prisons, particularly at the federal level. Following the 2024 U.S. presidential election, stock prices of for-profit prison companies surged; CoreCivic’s price increased by 55% while Geo Group’s grew 74%. For years, the annual meeting with Geo Group was in Boca Raton, Florida, but as of 2017, the meeting was changed to President Trump’s Miami golf club [27].

These companies made clear to shareholders that new contracts and opportunities would boom from the election results, especially with Immigration and Customs Enforcement (ICE) [28]. In addition, there is a connection between the former U.S. Attorney General Pam Bondi and private corrections, as her lobbying record includes work for GEO Group. This lobbying work, along with work for other governments, raised conflict of interest questions leading up to and during her confirmation hearing [29]. As a final indicator of forthcoming growth in private prisons, one of the first presidential executive orders of 2025 reversed the elimination of contracts between the U.S. Department of Justice (DOJ) and “Privately Operated Detention Facilities” [30] (para. 1).

Privatization, neoliberalization, and marketization are present throughout and beyond criminal justice and span treatment, housing, human resources/training, transportation, and educational programming [12], acute medical/health care, and long-term care (LTC), among other areas. Below, we draw parallels between LTC and prison systems, which may seemingly be quite contrasting and different landscapes. Yet, despite the different conditions in prisons and LTC, when we compare the privatization of the criminal justice context with the privatization of LTC, there are many similarities that emerge. For example, both contexts include sex/gender segregation, racialization, and involve structural violence. We feel that long-term care is the right comparator because it has similarities in the way prison systems are organized and structured. Broadly, long-term care often includes three sets of actors: the first are employees/workers, the second are residents, and finally the third, the structures that organize capital. Prison systems, as we described above, also include employees/workers, residents, and structures that organize capital.

LTC is one of the most contentious areas within health and social care, which is currently being debated in multiple contexts, such as management, delivery, financing, home-based vs. institutional care, staffing/workforce, and private versus public insurance, among other things. These contentions have also become more visible after the global COVID-19 pandemic and are expected to continue to experience significant changes as we experience an aging demographic in the United States and across the globe. In LTC, the private or corporate entities (major institutional holders) that are the largest shareholders include private equity firms [31]. These firms include Formation Capital which operates through Consulate Healthcare and Extendicare; Assured Healthcare Partners (formerly BlueMountain Capital) which operates Regency Integrated Healthcare Services; GI Partners which owns Plum Healthcare; Fillmore Capital Partners who own Golden LivinCenters; and McCarthy Capital who own Life Care Services, among others [32].

## 3. Theories and Schools of Thought Explaining the Trend of Privatization and Marketization in LTC

In LTC, privatization, neoliberalization, and marketization of services reflect micro-level interactions of people within the context of the market model, and also by the broad neoliberal ideologies and interests that subject care to the demands of individualization, profit-maximization, privatization, and cut-backs [7,33,34]. For example, a neoliberal model characterizes the application of market principles of supply and demand to the organization and function of care work [35] and often under the guise of efficiency and elimination of wasteful resources. “The term ‘neoliberalism’ was originally coined in 1938 to describe fairly moderate economic policies, consisting of a free market with competition, but supported by a strong and impartial state”, However, it has evolved to mean “[…] broad support for a capitalist, free market economy, and for a reduction in the regulatory power of the state” [7] (p. 422).

Like prison systems and carceral facilities, services in LTC facilities are often contracted out to control costs and include the adoption of for-profit business-oriented managerial techniques [36,37]. Under these circumstances, the neoliberal model of care narrowly conceptualizes care as quantifiable, physiological tasks that are counted, measured, and sold as if they are packaged products to consumers of care [38]. Again, this is similar to the conditions in the private or corporate carceral systems where “piecemeal privatization of functions, utilities, and services within state prisons make them operate more like private facilities, and public actors respond to the cost/benefit pressures of the market just like private ones” [7] (p. 411). To maximize profits, this market model focuses on cost-cutting, and this has troubling implications for workers [33]. It begins with reduced staffing, and work that relies upon both the paid and unpaid labor of poorly remunerated and low-status care workers, who are from various social locations marked by gender, race and class [39,40].

These work situations have translated to high workloads and workers’ exposure to health and safety hazards [33,41,42,43]. Researchers have identified that this neoliberal context results in a workplace struggle that materially coerces workers to perform unpaid care so that they retain their occupation, yet workers also feel a compulsion to work through moral principles of altruism, as one qualitative study across three different regions consisting of 83 interviews has demonstrated [44]. As a result, workers may self-identify themselves as being good and just when they provide services outside of their official contractual obligations [33,44], but realistically they are exploited through such moral principles. Under such contextual norms, the working conditions of such workers include structural and personal violence, and precariousness [45,46].

Although the populations served in LTC and PIC differ—older adults and people with disabilities in LTC versus incarcerated persons in correctional settings, respectively—workers in both sectors occupy structurally similar positions within neoliberal labor regimes. Indeed, labor precarity in LTC and corrections seems to reflect cost-containment strategies frequently manifest through understaffing, wage suppression, casualization of employment, increased surveillance, and intensified workloads. In LTC, these conditions disproportionately affect women, racialized workers, immigrants, and low-income employees whose labor has historically been undervalued because it is associated with caregiving and social reproduction [34,47,48]. In the PIC, workers similarly experience staffing shortages, mandatory overtime, heightened surveillance, and increased exposure to workplace hazards as institutions seek to maximize efficiency while minimizing expenditures [49]. Consequently, both sectors rely on forms of labor precarity characterized by employment insecurity, inadequate compensation, limited workplace autonomy, and chronic resource constraints. Importantly, labor precarity in LTC and the PIC is sustained not only through economic mechanisms but also through moral and ideological processes (to serve the public, to serve older adults). The resulting work environments expose employees to physical hazards, psychological strain, moral injury, and economic insecurity while simultaneously relying on workers’ sense of duty to sustain service provision. Examining these sectors together highlights how neoliberal policies produce similar patterns of labor exploitation across seemingly distinct domains of care and social control, revealing the broader political–economic conditions that generate precarious work in contemporary welfare and carceral institutions.

## 4. Costs of Confinement

Drawing a parallel to the criminal justice system, its privatization and marketization model alludes to the descriptions of what scholars describe as the 4 Ds of precarious work: dangerous, difficult, damned, or dirty [47,50,51]. As we explore the concept of precarious work in the literature, one of the more nuanced and recurring themes includes motivations to profit at the expense of workers’ health and wellbeing. Cost-control measures include reductions in staffing levels, yet contradict the expectation for expansion of prison ecosystems that, if funded, would otherwise lead to more revenue-generation:

“The unregulated private correctional institutions whose primary motives are profit are incentivized to seek it at all costs, and, with a lack of a strong state regulatory power over their operations, are likely to skimp and save on costly goods, services, and programs. As a result, there are serious concerns that conditions in private prisons will be worse, since unregulated private enterprises will maximize their profit-and their bloated executive pay-at the expense of the inmates. The state finds private greed difficult to regulate because citizens repeatedly vote down bond issues that fund prison expansion while at the same time demanding increases in incarceration”.

[7] (p. 424)

Researchers found that in the long-term, public prisons are less costly when compared to private (for-profit, corporate) prisons [52]. This analysis projects costs on a 25-year timeline that considers the shorter-term cost savings of using a for-profit company against their documented higher recidivism rates [52]. Aside from these established consequences of for-profit incarceration, costs that characterize incarceration irrespective of profit motivations also apply to this form of incarceration. Some of these notable consequences include social, financial, and emotional costs for families and children of incarcerated people [53]. One of the social consequences of policies driven by neoliberalism is that it has ballooned U.S. prisons, incarcerating largely the working and workless urban poor [54]. Another social consequence is that it creates two-tiered citizenship. For formerly incarcerated individuals, the concept of two-tiered citizenship in America is instructive. The incarceration literature highlights disparate impacts for some, notably poor Black Americans, where their social position is lowered, with profound impacts.

It is “[…] the notion of ‘custodial citizenship’: a second-class citizenship that transforms people into ‘custodial citizens’ through their interactions with public institutions such as the police or court system. It is through these mostly negative interactions that custodial citizens learn what their ‘place’ in society is. Upon internalizing the notion that they belong to an inferior citizenship group, custodial citizens withdraw from political life. […] when people are convicted of a crime, they are steered into ‘carceral citizenship,’ which is an alternate, but not necessarily inferior, legal status that carries its own unique set of restrictions, obligations, and benefits. Contact with the carceral state suppresses political engagement because it limits time, money, and opportunities to participate in the affairs of one’s community.”

[55] (p. 2)

Clearly, the social costs of incarceration for individuals and families are pronounced, but incarceration also comes with massive financial costs for people in the criminal legal system, their loved ones, and governments. It is estimated that justice-involved individuals and their loved ones spend almost $27 billion annually in various fines and fees related to their case [56]. In addition to this individual spending, corrections spending for public agencies in the US is more than $115 billion annually, a 27% increase since 2017 [56]. Given the massive financial costs associated with incarceration, this is a lucrative endeavor for the industry, similar to profitability seen in LTC. For example, in 2025, GEO Group revenues were $2.6 billion with a net income of $254.3 million [57]. In that same year, CoreCivic’s revenues were $2.2 billion, with net income of $116.5 million [58].

Due to high costs of America’s large prison system, the current federal administration recently announced a policy idea of sending American criminals who are repeat offenders to foreign jail and prison systems in order to save massive amounts of money because private, for-profit prisons “charge us a fortune” [59]. Although clearly this was only a policy idea, it reinforces the concept of two-tiered citizenship in America as we discussed above.

The administration is also seeking to cut the federal budget through President Trump’s newly created, and prematurely dissolved, Department of Government Efficiency (DOGE), which was led by billionaire Elon Musk, and boasted savings in government spending by slashing lease agreements, contracts, programs, and services, which is argued to be an illusion of efficiency [60,61,62]:

“DOGE backed a rapid restructuring drive built around a simple offer: resign and retain salary and benefits until the end of the fiscal year. It was marketed as voluntary and clean—a way to slim down without messy redundancies. A Senate investigation later reported that roughly 200,000 employees accepted the deal, costing about $14.8 billion in pay and benefits for months of work not done. The contradiction was clear. A project sold as an assault on waste spent billions paying people to leave, justified as future savings. Supporters framed it as a buyout: pay now, save later. But exits are not merely headcount reductions. They drain institutional memory, overload remaining teams, increase reliance on contractors, and raise the likelihood of mistakes”.

[61], (p. 27)

Such cutbacks on spending on American infrastructure align with the goals of liberal and neoliberal ideologies, as indicated earlier, aimed at profit-maximization, privatization, and the application of market principles of supply and demand, in efforts to save money under broadly implemented measures of efficiency and elimination of wasteful resources. However, the reality is that these cutbacks can have adverse consequences, as we have already described.

## 5. Immigrant Detention and For-Profit Corporate Incarceration

Immigration detention is important to consider in any discussion of private corrections given the contours of illegal immigrant detention in the U.S. and the significant place that immigrant detention occupies for the profits of private corrections companies. The U.S. operates the world’s largest immigrant detention system and requires non-governmental, often private companies to carry out its operations [21]. Importantly, however, immigration detention is different from prison detention. Immigrant detention is “the practice of incarcerating noncitizens who are apprehended” and keeping these individuals “in custody until they are released, bonded and paroled, or deported from the United States” [63] (p. 532). Though there are similarities to criminal incarceration, immigration detention is a type of civil confinement [64], and it is often carried out for indefinite periods of time compared to prison detention. Consequently, many constitutional protections that are available under criminal law do not exist in the immigrant detention system [65]. Furthermore, immigrant detention entails administrative processes and procedures, with no federal right or government-appointed lawyers as legal representation for immigrant detainees, whereas prison detention is intended as a punitive system and has access to legal representation [65]. However, they heavily intersect, with many immigrants held in the same prison-like environments and directly transferred from local jails to detention centers [65]. Overall revenues of the private, corporate corrections industry have increased significantly in this space as this form of detention has soared in recent years [66,67]. To support the sprawling immigrant detention system, Immigration and Customs Enforcement (ICE), the federal agency responsible for enforcing migration policy in the U.S., must outsource bedspace since ICE owns and operates just a few facilities. Accordingly, it contracts with private companies and local jails to house detainees [64] which are often massive monetary commitments. For example, in 2023 Geo Group had revenues from ICE of $1 billion (which was 43% of their total revenue) [68,69]. The second largest contractor, CoreCivic, also had large 2023 revenues generated from ICE detentions at $552 million, which was 30% of their total revenue [69]. Immigrant detention is now a core focus of their business, making the heavy reliance on companies such as GEO Group and CoreCivic a reciprocally beneficial relationship:

“In recent years, roughly two-thirds of ICE detainees have been held in facilities operated by private firms […]. As the U.S. government has become reliant on private companies to detain immigrants, so too have private companies become reliant on the U.S. government to supply immigrant detainees”.

[21] (p. 1)

It is argued that this business model of a private, for-profit system seeks to expand and encompass new fields, new markets, and new profits, such as the undocumented detention market [7]. Some have labeled this as “crimmigration” [7] (p. 424), or the criminal management of immigration, which has increased as a response to market conditions, and not merely as a response to concerns about terrorism or undocumented migration [70]. Importantly, the private sector has been used for immigrant detention in the U.S. for many years. Despite federal policy shifts in recent years regarding the use of private prisons, such as the executive orders referenced previously which ended federal use of private prisons (under the Biden Administration) and then reintroduced it (under the Trump Administration), these orders did not pertain to immigrant detention, which has relied heavily on private or corporate corrections for many years [30].

The current federal administration has pledged to crack down on illegal immigration, largely predicated on the myth that such migrants commit more crimes than domestically born persons [71]. The administration also recently announced that 956 people were arrested in just a single day on 26 January 2025 [72], and further arrests were made the following days, with immigration raids conducted in major cities across the U.S., such as New York, Los Angeles, and Philadelphia. Problematically, immigration raids often conflate the civil offenses of being an undocumented migrant and crossing the border as a criminal offense and having prior criminal records, when in fact, very few undocumented workers have any criminal background and many have crossed legally but may have overstayed their visa. To further complicate matters, the current administration refuses to differentiate between migrants with criminal histories and those without criminal histories who are in custody [71].

Once confined, those swept up in the immigration detention system often face problematic conditions. Research shows that conditions of confinement are worse in immigrant detention facilities compared to other carceral settings. Studies have found that there are higher levels of understaffing and detainee overcrowding [65,66], and detainees frequently experience problematic use of segregation, insufficient medical care, labor exploitation, and physical and sexual abuse [64,66].

Immigrant status is just one area where disparity exists in the broader prison landscape, including within non-governmental, often private facilities. One of the visible patterns of the American prison system is that it has a high concentration of impoverished people who are predominantly from non-white communities, which reflects historical, social, and economic conditions as well as policies having racial implications [73]. This disparity also means there are health inequities in these communities. For example, the health and safety of incarcerated persons, who are often racialized, are also at a high risk of mental health issues, type II diabetes, HIV/AIDS, tuberculosis, and COVID-19 compared to the general public and medically underserved [8,71], although this may depend on private or public provider and other factors. Accordingly, the present realities of mass incarceration in America’s prison system reflect internal contradictions and crises of structural discrimination and neoliberal forms of capitalist accumulation.

## 6. Privatization and Marketization from a Commercial Determinants of Health (CDoH) Perspective

Prison system privatization and marketization seem to be reflective of a broader trend that has parallels to privatization and marketization in health and social care. While private, for-profit LTC and prison systems differ in purpose, legal status, and populations served, they share similar logics of marketization, labor precarity, and under-resourcing. For example, in long-term care, private and for-profit management has led to the increased burden on work being carried out by fewer and fewer employees, which results in incomplete care, gaps in care, and poor outcomes among residents [34,74].

The hundreds of thousands of prisoners currently incarcerated in the U.S. have a constitutional right to healthcare [54]. For-profit, corporate, and private prisons, like privatized and marketized health and social care systems, though, tend to have lower quality health outcomes. Studies have shown that private prisons have increased workloads that burden workers and also have poorer outcomes among residents, including increased morbidity and mortality [8,54]. We summarize similarities and differences between these types of prisons and long-term care facilities in Table 1.

Proponents of privatization and marketization of LTC and prison systems have argued that private, corporate facilities both operate more cheaply and with higher levels of performance [52,75,76,77,78]. However, research shows that compared to public facilities, private facilities tend to have higher violence, misconduct, resident wandering or escape, lower or unmet standards of care, and systemic problems in maintaining and securing facilities as well as issues in compliance and monitoring [52,77,78].

In the prison system, ironically, the healthcare needs of correctional populations are protected by the U.S. Constitution, as asserted previously:

“Indeed, the historiography of prison healthcare often begins in the courts with the landmark 1976 Supreme Court case Estelle v. Gamble. In 1973, J.W. Gamble, a prisoner at the Texas State Penitentiary in Huntsville, sued his prison doctor, the warden, and the Texas Department of Corrections for cruel and unusual punishment. While the Court ultimately ruled against Gamble, they affirmed that ‘prisoners [must] be provided with medical care and that deliberative indifference by prison personnel to a prisoner’s serious illness or injury violates the Eighth Amendment’ (1976: n.p.)”.

[54] (p. 11)

In light of the range of motivations, problems, and outcomes surrounding private carceral institutions, we argue that private correctional facilities, including those for immigrant detention, should be understood as a commercial determinant of health (CDoH). These determinants are defined as “strategies and approaches used by the private sector to promote products and choices that are detrimental to health” [79] (p. e895). This idea has been applied to a variety of industries responsible for negative health outcomes, such as alcohol, tobacco, and processed foods [79,80], but it has also been used to consider health problems in other spaces. Most notably, the PIC is described as a CDoH and documents the negative health outcomes that coexist with mass incarceration in the U.S. [6]. In addition, there are calls for an increased understanding of disproportionate health outcomes and a closer examination of the policy influence of the PIC [6]. This analysis fills that gap by specifically tracing the expansion of the private carceral apparatus in the U.S., which, as previous studies have found, comes with greater health challenges for those in custody. Private prisons have been found to have fewer programs for HIV/AIDS treatment, mental health, and substance abuse [81]. Significantly, health care is often privatized in prison settings, regardless of whether the prison is privately or publicly managed [82].

As noted previously, carceral facilities do not exist in isolation:

“The health of persons living and working in those settings reverberates through communities across the country in myriad ways—when staff return home from work each day, when incarcerated persons are transferred between facilities in different jurisdictions or participate in work release programs, and when most eventually are released to reunite with their families”.

[8] (pp. S2–S3)

Given the above example recognizing the interconnectedness of staff, we emphasize that not only workers, families and communities, but also residents and the environments in which carceral facilities are situated are a part of the larger micro, meso, and macro contexts that are impacted by these organizations and structures. For instance, firms that have for-profit, private or investor-owned [83] structures are accountable to shareholders and profits are redistributed to them. This is because shareholders own the corporation and […] the sole responsibility of the corporate manager is to “increase profits” [84] (p. 55). Corporations, however, have “scaled back dividend distributions and re-invested more of their excess profits into their operations” [84] (p. 27) due to capital demands that “drove corporate enterprises to re-invest a greater proportion of their earnings into operations rather than make distributions to shareholders” [84] (p. 20).

However, quality metrics between private, for-profit, or investor-owned versus public and/or not-for-profit systems are different, since a greater proportion of profits are reinvested to improve organizational function, quality, and services in public and nonprofit systems. Indeed, research confirms that non-profit LTC has higher quality processes, including processes in areas of patient focus and areas of responsiveness where there is a corresponding association with outcome quality, with the exception of leadership roles, e.g., director of nursing [85]. The reasons for higher quality in public or not-for-profit systems is explained by a number of factors, including more capital reinvestment available because not-for-profit organizations do not pay taxes on income, property, or purchases; leadership renumeration is higher in for-profit, corporate, or investor-owned systems [86] and lower in not-for-profit systems which softens incentives and reassures higher quality [87,88]; as well as higher administrative costs in the former compared to the latter [84]. Not-for-profit firms have less financial incentive to cut wages, and may offer better working environments for employees as well as improvement in quality of the product, whereas for-profit firms have strong leadership and shareholder incentives that lead to inefficient behavior resulting from profit maximization and encourage shirking on quality [89]. For example, for-profit LTC evidently used more sedatives (a cheap way to keep patients calm) than the non-profit LTC which is a cost-reducing strategy adversely affecting quality [90]. “Private prisons might abuse prisoners by hiring cheaper guards and failing to train them, private hospitals may refuse to treat patients on whom hospitals generally lose money, […], and so on [87] (p. 138). Accordingly, reframing the success in corporate and often private facilities needs to be re-examined with these additional contexts and included in avenues of future research.

## 7. Conclusions

In this paper, we have used an interdisciplinary perspective to compare the for-profit, neoliberal prison system with the for-profit, neoliberal long-term care system. Our main contribution to the literature is that neoliberalization of these organizations and structures operates as a shared political–economic logic across care and carceral systems, with distinct but related health consequences. As this analysis has shown, privatization and marketization in corrections—particularly through for-profit prisons and immigrant detention—reflect the broader neoliberal trend of shifting public responsibilities to private, corporate actors under the guise of efficiency and cost reduction. Yet, this pursuit of profit often comes at the expense of human dignity, equity, and health. The parallels between privatization and marketization in the criminal justice system and in long-term care (LTC) reveal shared logics that prioritize financial returns over social well-being. Both sectors rely heavily on underpaid and overburdened labor forces, operate within gendered and racialized hierarchies, and produce environments where structural violence and neglect are normalized as cost-saving measures. One of the limitations of this paper is that while it is grounded in existing criminology, public health, and political economy literature, the comparison is suggestive rather than explicit, and empirical evidence gathered through further interdisciplinary studies would be beneficial.

Importantly, when viewed through the lens of the Commercial Determinants of Health (CDoH), corporate/private prisons and correctional services can be understood as entities that perpetuate and exacerbate health inequities, which are also paralleled in neoliberal, for-profit long-term care services. These institutions not only shape the health outcomes of their residents and staff—who are disproportionately poor and/or racialized—but also affect the wellbeing of families and communities beyond their walls. For-profit privatization, neoliberalization and marketization therefore extend harm through social, economic, and health-impacting pathways, reinforcing cycles of disadvantage and precarity. Accordingly, the U.S. reliance on privatization within its carceral and care systems reveals deep ideological commitments to neoliberal efficiency rather than to justice, equity, or care.

Our work has several implications. First, the U.S. reliance on privatization and marketization within its carceral and care systems needs to be re-evaluated through an interdisciplinary lens—bridging criminology, public health, and social policy. Secondly, the fields of criminal justice and public health need to continue to effectively collaborate, share data, and support both workers and residents in their settings while challenging policymakers, scholars, and practitioners to reconsider the ethical and structural consequences of profit-driven governance. Meaningful reform and strengthening of publicly held models will require reasserting the public responsibility to provide humane, equitable, and accountable systems of care—both within correctional institutions and across society at large.

## Figures and Tables

**Table 1:** Comparison of features often present in prisons and long-term care facilities in the United States.

Features	Long-Term Care Systems	Prison Systems
Worker Contexts	− Large numbers of front-line workers; − Administrative staff exist; − Workers provide activities of daily living (ADLs); − Workers consist of nurses, personal support workers, dietary aides, and so forth; − Limited rehabilitative staff, therapists, recreational staff; − Limited career mobility for front-line workers; − Precarious work, especially in neoliberal, for-profit (and often private) types.	− Large number of front-line workers; − Administrative staff exist; − Workers provide correctional services (security-focused); − Workers consist of officers and surveillance staff; − Limited rehabilitative staff, counselors, medical staff, and so forth; − Limited career mobility for front-line workers; − Precarious work, especially in neoliberal, for-profit (and often private) types.
Main Goals	− Accommodate residents (~1.3 million); − Surveillance using technology, locked units for high-risk residents.	− Accommodate residents (~1.3 million) − Security; − Surveillance using technology, locked units.
Profit Structures	− For-profit structures incentivize cost-cutting; − Under-resourcing, e.g., understaffing, low pay are common features, especially in neoliberal, for-profit (and often private) types.	− For-profit structures incentivize cost-cutting; − Under-resourcing, e.g., understaffing, low pay are common features, especially in neoliberal, for-profit (and often private) types.
Gender and Race Contexts	− Predominately female workforce; − High reliance on immigrant and minority women; − Predominately older adults (mostly women and people with disabilities).	− Predominately male workforce; − Disproportionate incarceration of minorities (African Americans, Hispanic/Latin Americans).
Risks	− High-risk environment; − Trauma, violence exposure; − High stress; − Physically demanding; − Emotional labor; − High job demand, low control.	− High-risk environment; − Trauma, violence exposure, and isolation; − High stress; − Physically demanding; − Emotional labor; − High job demand, low control.

## Data Availability

No new data were created or analyzed in this study. Data sharing is not applicable to this article.

## Abbreviations

The following abbreviations are used in this manuscript:

LTC	Long Term Care
PIC	Prison Industrial Complex
